# Sublethal chlorpyrifos exposure induces intergenerational fitness costs and mitochondrial alterations in *Blattella germanica*

**DOI:** 10.1016/j.cris.2026.100136

**Published:** 2026-10-23

**Authors:** Xin Li, Jiang-Ling Chen, Zi-Han Xu, Jun Tao, Yi Chen, Shu-Lin He, Fei Li, Cao Zhou

**Affiliations:** Chongqing Key Laboratory of Vector Control and Utilization, Institute of Entomology and Molecular Biology, Chongqing Normal University, Chongqing, China

**Keywords:** *Blattella germanica*, Intergenerational effects, Mitochondrial dysregulation, Detoxification response, Reproductive fitness cost, Age-stage, two-sex life table

## Abstract

Under real-world conditions, the German cockroach *Blattella germanica* is frequently exposed to sublethal insecticide concentrations, yet the long-term consequences for population dynamics remain poorly understood. Here, we investigated the intergenerational effects of sublethal chlorpyrifos exposure in this urban pest. Newly emerged adults were treated with LD_10_ or LD_30_, and fitness-related traits and molecular responses were evaluated in the F_0_ and F_1_ generations. Sublethal exposure reduced fecundity and longevity in the F_0_ generation. Parental exposure also induced intergenerational effects in the F_1_ generation, including prolonged development, reduced survival, altered reproductive timing, and a decreased net reproductive rate. Population projection analysis indicated that LD_10_ suppressed long-term population growth. Chlorpyrifos further induced dose-dependent and intergenerational changes in detoxification-related genes, particularly *BgCYP6ER1* and *BgCYP6J1*. In addition, mtDNA copy number increased under LD_30_, whereas *BgCytb* transcription was downregulated in the F_1_ generation, suggesting mitochondrial dysregulation under sublethal stress. These findings demonstrate that sublethal chlorpyrifos exposure can induce intergenerational effects on fitness-related traits and molecular responses. and highlight potential implications for pesticide risk assessment and resistance management.

## Highlights

• Sublethal chlorpyrifos exposure caused intergenerational effects on fitness-related traits.
• Parental chlorpyrifos exposure prolonged F_1_ male developmental duration and altered offspring survival and reproduction.
• Parental chlorpyrifos exposure influenced projected population growth of offspring.
• Detoxification genes showed dose- and generation-dependent expression patterns following chlorpyrifos exposure.
• Mitochondrial alterations were associated with sublethal chlorpyrifos stress responses.

## Graphical abstract

**Figure:**
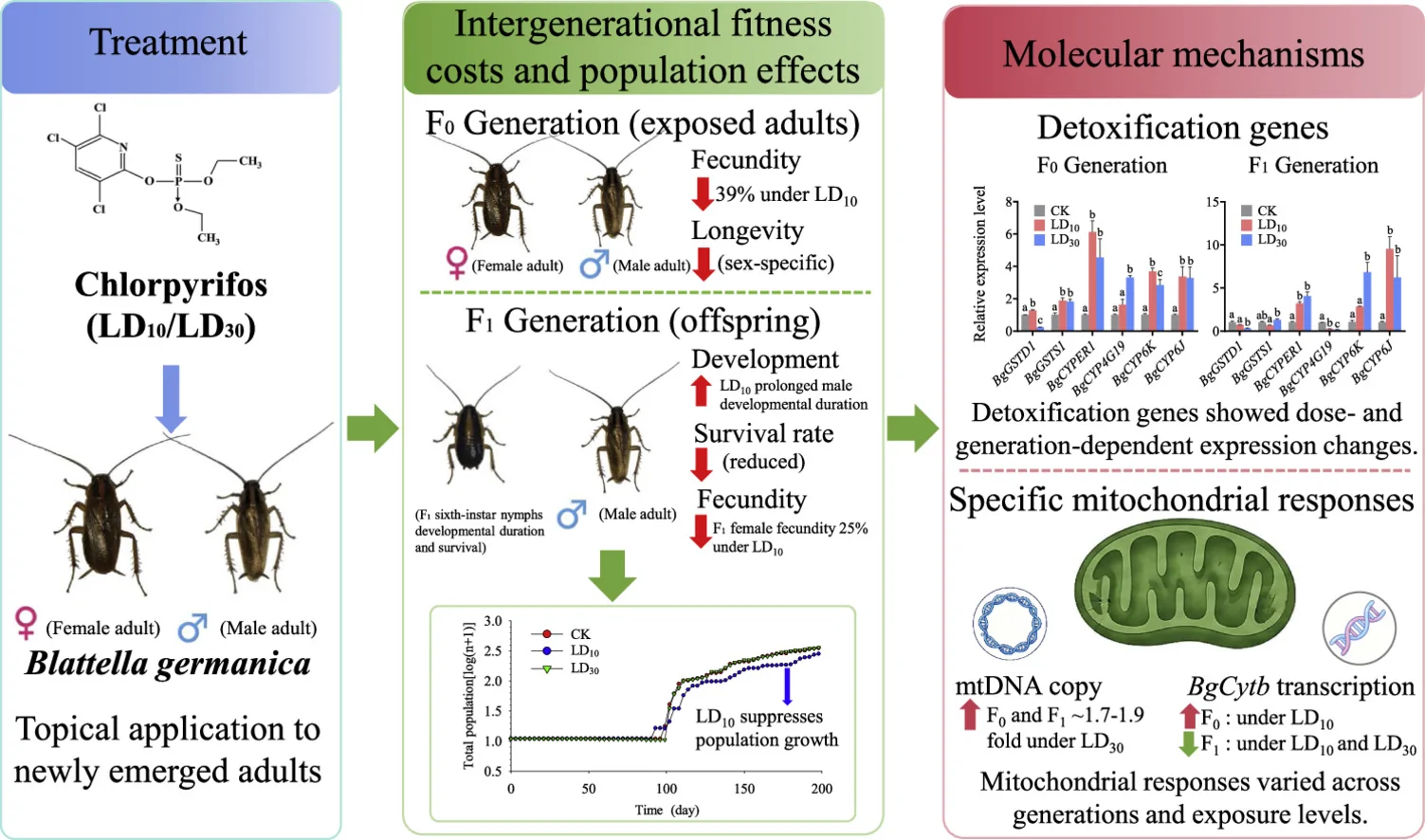

## 1 Introduction

The German cockroach, *Blattella germanica*, is one of the most important urban public health pests, characterized by high reproductive potential, broad dietary adaptability, and cryptic behavior (Rust & Reierson, 1991). It not only acts as a mechanical vector of various pathogenic microorganisms (Menasria et al., 2014) but also produces potent allergens that are strongly associated with allergic diseases and asthma (Do et al., 2016), posing a significant threat to public health. Despite advances in integrated pest management (IPM), chemical insecticides remain the primary means of controlling its populations (Cochran, 1995; Zhu et al., 2016). Under field conditions, however, insecticides are often rapidly degraded by environmental factors, resulting in widespread exposure of insects to sublethal concentrations (Desneux et al., 2007), the ecological consequences of which remain insufficiently understood.

Sublethal insecticide exposure can markedly affect insect development, reproduction, survival, and population dynamics (Müller, 2018). Among these effects, fitness costs are considered a key mechanism linking individual-level responses to population dynamics, reflecting trade-offs in resource allocation between detoxification and life-history processes such as growth and reproduction (Ffrench-Constant & Bass, 2017; Kliot & Ghanim, 2012). Previous studies have shown that chlorpyrifos induces developmental delays, reduced fecundity, and suppressed population growth in various insect species (Garlet et al., 2021; Wang et al., 2017). Sublethal insecticide exposure can also impair parental fitness by altering key life-history traits, including survival and reproduction (Afza et al., 2023; Margus et al., 2019). Such effects in the directly exposed parental generation may subsequently extend to subsequent generations (Bonduriansky & Day, 2009). However, evidence for the fitness consequences of chlorpyrifos exposure in *B. germanica* remains inconsistent. Some studies have reported hormesis-like responses following exposure to low sublethal doses of chlorpyrifos (approximately 0.05-0.08 μg/insect, corresponding to LD_10_ levels), including increased longevity or fecundity (Abd-Elghafar et al., 1992), whereas others have documented reproductive suppression and developmental retardation (Hamilton & Schal, 1990). These discrepancies highlight the complexity of fitness responses to sublethal stress in this species, and whether such exposure consistently results in fitness costs and intergenerational effects remains to be clarified.

At the molecular level, insect adaptation to insecticides is largely mediated by detoxification enzyme systems, particularly cytochrome P450 monooxygenases and glutathione *S*-transferases (GSTs) (Feyereisen, 1999; Li et al., 2007). Chlorpyrifos exposure induces the upregulation of these enzymes; however, reactive oxygen species (ROS) generated during P450-mediated metabolism can lead to oxidative stress and mitochondrial dysfunction (Liu et al., 2015). Mitochondrial function is closely associated with cellular energy metabolism and stress responses and can be evaluated through indicators such as mtDNA copy number and transcriptional activity (Montier et al., 2009; Rodrigues et al., 2022), both of which often exhibit compensatory responses under environmental stress (Zeng et al., 2024). Nevertheless, whether chlorpyrifos-induced fitness costs are mediated through a pathway involving detoxification activation, ROS accumulation, and mitochondrial dysfunction remains unclear, particularly in *B. germanica*, where direct evidence is lacking.

Against this background, the present study employs *B. germanica* as a model to investigate the fitness costs and underlying molecular mechanisms associated with sublethal chlorpyrifos exposure. Specifically, the effects of LD_10_ and LD_30_ on survival and reproduction in the F_0_ generation were assessed, and age-stage, two-sex life tables were constructed for the F_1_ generation to evaluate population dynamics. In addition, the expression of detoxification-related genes (P450 and GST) and a mitochondrial marker gene (*BgCytb*), along with mtDNA copy number, was quantified. By integrating population- and molecular-level analyses, this study aims to elucidate the dose-dependent and intergenerational effects of sublethal chlorpyrifos exposure, thereby providing a theoretical basis for optimizing pest management strategies and assessing resistance risk.

## 2 Materials and methods

### 2.1 Insects and bioassay

The *Blattella germanica* colony was obtained from Prof. Zongqing Wang (Southwest University, China) (Shi et al., 2026). Cockroaches were maintained in an artificial climate chamber (LRG-380B) at 25 ± 1°C, 50-80% relative humidity under constant darkness, with commercial rodent diet and water provided *ad libitum*. Newly emerged adults (<24 h old) of both sexes were used throughout the experiments.

Technical-grade chlorpyrifos (95% purity; Shandong Xingniao Biotechnology Co., Ltd., China) was dissolved in acetone to prepare five concentrations (0.05, 0.1, 0.15, 0.2, and 0.4 μg/μL). Toxicity was assessed using a topical application bioassay following the GB/T 26352-2010 guideline. After CO_2_ anesthesia, healthy and uniform adult males were selected, and 1.0 μL of each solution was applied to the ventral thoracic sternum between the second and third coxae. Five independent biological replicates were performed for each concentration, with 10 males per replicate (n = 50 per concentration). An acetone-treated control group was included, comprising five biological replicates of 10 males each (n = 50), and control insects underwent the same anesthesia and topical application procedures, receiving 1.0 μL acetone without chlorpyrifos. Mortality was recorded at 72 h, and the dose-mortality relationship was used to estimate LD_10_ and LD_30_ (SM1.1; Table S2).

### 2.2 Assessment of development and reproduction in the F_0_ and F_1_ generations

Based on the bioassay results, LD_10_ and LD_30_ were selected as sublethal doses. Newly emerged adults (<24 h old) were randomly selected and treated with acetone, LD₁₀, or LD₃₀ using the topical application procedure described above. Three biological replicates were established for each treatment. Sufficient numbers of adults were treated to ensure that, after treatment-related mortality, 10 surviving female–male pairs were obtained for each replicate. Females and males were maintained as pairs at a 1:1 ratio, with food and water provided ad libitum. Adult longevity was recorded daily from adult emergence until death. Oothecae were monitored daily for developmental progression, hatching status, and hatching time. Fecundity was defined as the total number of eggs produced by each female throughout her lifetime, including both hatched and unhatched eggs.

Sixty F_1_ nymphs from each treatment were individually reared under the same conditions. Molting, developmental duration of each of the six nymphal instars (N1-N6), and survival were recorded daily until adult emergence. Newly emerged F_1_ virgin females and males were paired within 24 h of emergence to assess adult reproductive performance, including the adult preoviposition period (APOP), total preoviposition period (TPOP), and fecundity. F_1_ fecundity was assessed using the same procedure as in F_0_. Adult survival in both the F_0_ and F_1_ generations was monitored daily from adult emergence until death. Individuals not used for reproductive assessment were retained for molecular analyses.

### 2.3 Insecticide exposure and molecular analysis

For both the F_0_ and F_1_ generations, adult females from the CK, LD_10_, and LD_30_ treatment groups were sampled at 6 days post-emergence. Three independent biological replicates were used for each treatment, with two females collected from each replicate (n = 6 females per treatment per generation). Sample were immediately frozen in liquid nitrogen and stored at −80°C until analysis. Each sample was divided for genomic DNA and total RNA extraction.

Genomic DNA was extracted using a commercial kit (CoWin Biosciences, China) and used to estimate relative mtDNA copy number by RT-qPCR based on the ratio of mitochondrial *BgCytb* to nuclear *BgEF1α* (Behura & Lobo, 2021). Total RNA was extracted using the HP Total RNA Kit (Thermo Fisher Scientific, USA), reverse-transcribed into cDNA, and used to quantify *BgCytb* transcription by RT-qPCR (Barshad et al., 2018). The expression of detoxification-related genes (*BgCYP6K1, BgCYP6J1, BgCYP6ER1, BgGSTd1, BgGSTs1*, and *BgCYP4G19*) was also quantified by RT-qPCR using *BgEF1α* as the reference gene. Reactions were performed using SYBR Green qPCR Mix (Siding Biotechnology, China) on a Bio-Rad CFX96 Real-Time PCR Detection System (SM1.3). Relative gene expression was calculated using the 2^−ΔΔCt^ method, with three technical replicates per sample. Primer sequences and amplification efficiencies are provided in Table S1.

### 2.4 Data analysis

Toxicity data were analyzed by probit analysis in SPSS 27.0 (Finney, 1978) to estimate LD_10_ and LD_30_. For the F_0_ generation, fecundity was analyzed using a negative binomial generalized linear mixed model (GLMM), with chlorpyrifos treatment as a fixed effect and biological replicate as a random effect, followed by Tukey-adjusted pairwise comparisons. Adult longevity was analyzed using Cox proportional hazards regression with biological replicate as a shared frailty term. Model diagnostics for the F_0_ fecundity and longevity analyses are provided in Supplementary Materials (SM1.4; Tables S6–S7).

For the F_1_ generation, age-stage, two-sex life-table parameters, including APOP, TPOP, net reproductive rate (*R*_0_), intrinsic rate of increase (*r*), finite rate of increase (*λ*), and mean generation time (*T*), were calculated using TWOSEX-MSChart (Chi & Liu, 1985; Chi, 1988; Chi et al., 2022). Definitions and calculation procedures for the reproductive and life-table parameters are provided in Supplementary Methods SM1.2. Standard errors were estimated from 100,000 bootstrap resamplings, and treatment differences were assessed using the paired bootstrap test. Population dynamics over 200 days were projected using TIMING-MSChart (Chi, 1990; Chi et al., 2023).

For F_0_ and F_1_ molecular data, relative expression levels and mitochondrial DNA copy numbers were analyzed separately for each gene or indicator using one-way ANOVA followed by Tukey’s multiple-comparison test. Statistical significance was set at *P* < 0.05.

## 3 Results

### 3.1 Effects of sublethal chlorpyrifos exposure on fecundity and longevity in the F_0_ generation

The toxicity regression equation for chlorpyrifos was Y = 4.00X + 3.10, with LD_10_ and LD_30_ values of 0.08 and 0.12 μg per individual at 72 h, respectively (Table S2). In the F_0_ generation, fecundity was significantly reduced following LD_10_ compared with CK (negative binomial GLMM: Wald χ² = 10.919, df = 2, *P* = 0.0043; Tukey-adjusted *P* = 0.0042), whereas fecundity did not differ significantly between LD_30_ and CK (*P* = 0.1224) (Table 1; Tables S3-S4). Treatment effects on adult longevity also differed between sexes. In males, LD_10_ significantly increased mortality risk compared with CK (HR = 2.59, *z* = 3.34, *P* < 0.001), whereas LD_30_ did not significantly increase mortality risk (*P* = 0.111). In females, LD_30_ significantly increased mortality risk (HR = 2.14, *z* = 2.66, *P* = 0.008), whereas LD_10_ had no significant effect (*P* = 0.126) (Table 1; Table S5).

**Table 1:** Fecundity and longevity of F_0_*Blattella germanica* adults following sublethal chlorpyrifos exposure.

Treatment	Fecundity (*F*)	Male longevity	Female longevity
CK	82.73 ± 5.37a	123.77 ± 5.44a	165.80 ± 7.34a
LD_10_	50.23 ± 7.46b	99.13 ± 4.56b	146.16 ± 8.40ab
LD_30_	62.93 ± 7.05ab	110.30 ± 6.19ab	134.03 ± 8.98b

Values are presented as mean ± SE. Different lowercase letters within the fecundity column indicate significant differences among treatments based on Tukey-adjusted pairwise comparisons following a negative binomial GLMM (*P* < 0.05). Different lowercase letters within the male and female longevity columns indicate significant differences among treatments based on pairwise comparisons from Cox proportional hazards models (*P* < 0.05). Biological replicate was included as a shared frailty term in the Cox models. CK, acetone-treated control.

### 3.2 Intergenerational effects on development, reproduction, and population parameters in the F_1_ generation

Parental sublethal exposure affected several developmental and reproductive traits in the F_1_ generation. LD_10_ significantly prolonged male development, mainly due to extended egg duration and prolonged first-, fourth-, and sixth-instar nymphal stages, resulting in a longer total pre-adult developmental duration (Tables S8-S9). In contrast, LD_30_ caused only limited, stage-specific effects on development. Female fecundity was significantly reduced under LD_10_, by approximately 25% relative to CK. APOP was significantly longer under LD_30_ than under LD_10_ but did not differ significantly from CK, whereas TPOP did not differ significantly among treatments (Table 2). The net reproductive rate (*R*_0_) decreased under both sublethal treatments, whereas the mean generation time (*T*) tended to increase under LD_10_. However, neither *R*_0_ nor *T*, nor the intrinsic rate of increase (*r*) or finite rate of increase (*λ*), differed significantly among treatments (Table 2).

**Table 2:** Reproductive traits and population parameters of F_1_*Blattella germanica* following parental exposure to sublethal chlorpyrifos.

Treatment	Adult preoviposition period (APOP) (d)	Total preoviposition period (TPOP) (d)	Fecundity (*F*)	Finite rate of increase (*λ*)	Net reproductive rate (*R*_0_*)*	Intrinsic rate of increase (*r*)	Mean generation time (*T*)/d
CK	17.70±1.90ab	109.04±1.95a	96.07±5.04a	1.0273±0.0013a	43.23±6.57a	0.2702±0.0013a	139.39±3.12a
LD_10_	15.19±0.87b	111.33±1.98a	72.63±9.04b	1.0242±0.0014a	32.68±6.15a	0.2393±0.0016a	145.70±3.72a
LD_30_	20.96±2.47a	114.50±2.64a	91.86±5.34ab	1.0275±0.0017a	42.87±6.41a	0.2711±0.0013a	138.52±2.59a

Values are presented as mean ± SE. Different letters within the same column indicate significant differences among treatments based on paired bootstrap tests with 100,000 bootstrap resamplings (*P* < 0.05). CK indicates the acetone-treated control group.

### 3.3 Effects on survival structure and population dynamics

Age-stage-specific survival rate (*s_xj_*) analysis showed that sublethal chlorpyrifos exposure reduced nymphal survival and increased developmental asynchrony, with effects intensifying at higher concentrations. Adult emergence was delayed in treated groups, accompanied by reduced overall survival (Fig. 1A, C, E). Age-specific survival rate (*l_x_*) further indicated that control populations maintained relatively stable survival up to approximately 150 days, whereas treated groups exhibited a marked decline at 100-120 days, suggesting increased early mortality (Fig. 1B, D, F). Reproductive output was significantly suppressed in treated groups. Both age–stage-specific fecundity (*f_x_*_8_) and age-specific fecundity (*m_x_*) showed reduced peak values (Fig. 1 B, D, F).

**Fig. 1:**
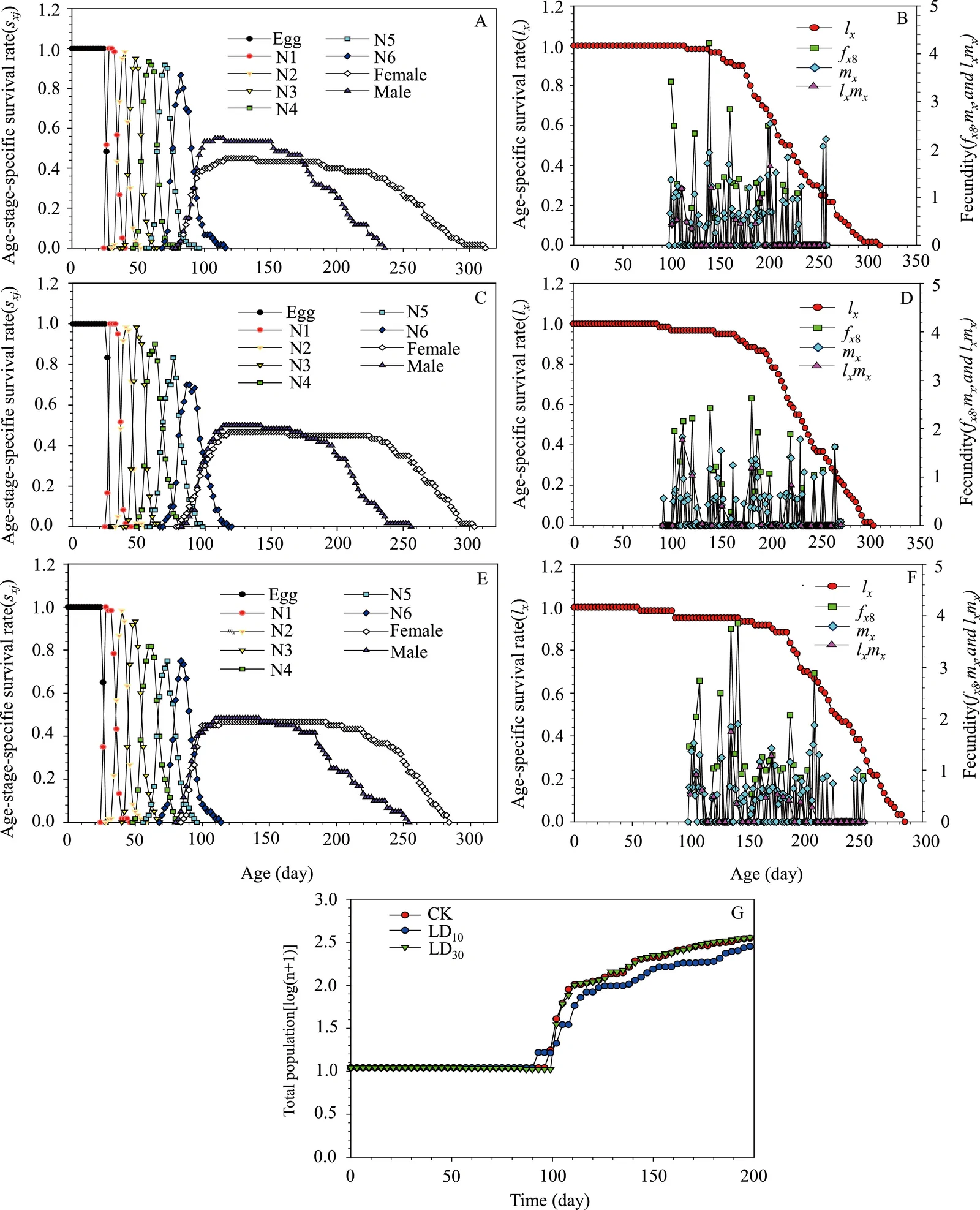
Age-stage-specific survival rate (*s_xj_*), age-specific survival rate (*l_x_*), age-stage-specific fecundity (*f_x_*_8_), age-specific fecundity (*m_x_*), age-specific net maternity (*l_x_m_x_*), and projected population dynamics of the F₁ generation of *Blattella germanica* following sublethal chlorpyrifos exposure. A-C show the age-stage-specific survival rate (*s_xj_*) for the control (CK), LD_10_, and LD_30_ treatment groups, respectively. N1: first-instar nymph; N2: second-instar nymph; N3: third-instar nymph; N4: fourth-instar nymph; N5: fifth-instar nymph; N6: sixth-instar nymph. D-F show the age-specific survival and fecundity parameters (*l_x_, f_x_*_8_, *m_x_*, and *l_x_m_x_*) for the control, LD_10_, and LD_30_ treatment groups, respectively. G shows the projected population dynamics over 200 days under different treatments.

Reproductive value (*v_xj_*) analysis indicated that LD_10_ advanced the timing of the reproductive peak but reduced its magnitude, whereas LD_30_ maintained relatively higher peak values but markedly shortened the effective reproductive period (Fig. S1). Life expectancy (*e_xj_*) declined with age in all groups, with females consistently higher than males. LD_10_ extended the maximum observed lifespan, whereas LD_30_ shortened the lifespan range, showing a clear dose-dependent pattern (Fig. S2). Population projections over 200 days indicated that LD_10_ exposure resulted in a sustained reduction in population size compared with the control, with the suppressive effect becoming increasingly pronounced over time (Fig. 1G, Fig. S3). In contrast, LD_30_ exposure altered the timing and rate of population growth, though these differences from the control gradually diminished over time, ultimately yielding a final projected population size comparable to that of the control group.

### 3.4 Effects on detoxification gene expression and mitochondrial dynamics

In the F_0_ generation, LD_10_ significantly upregulated cytochrome P450 genes, including *BgCYP6ER1* (∼6.1-fold), *BgCYP6K1*, and *BgCYP6J1*, while slightly inducing *BgGSTs1*. In contrast, LD_30_ strongly induced *BgCYP4G19* and maintained elevated expression of multiple P450 genes, but significantly suppressed *BgGSTd1*. In the F_1_ generation, distinct transcriptional responses were observed. *BgCYP6J1* was markedly upregulated under LD_10_ (∼9.6-fold), whereas *BgCYP6K1* and *BgCYP6J1* remained highly expressed under LD_30_. By contrast, *BgCYP4G19* showed a decreasing trend under both treatments, and *BgGSTd1* exhibited a similar downward trend, with significant downregulation observed only under LD_30_ (Fig. 2A, D), indicating intergenerational transcriptional responses. At the mitochondrial level, *BgCytb* expression and mtDNA copy number exhibited dose-dependent patterns. In the F_0_ generation, *BgCytb* expression was significantly upregulated under LD_10_ but showed no significant change under LD_30_ (Fig. 2B, C). In the F_1_ generation, *BgCytb* expression was significantly downregulated under both treatments. Meanwhile, mtDNA copy number increased significantly (approximately 1.8-1.9-fold) under LD_30_, whereas no significant change was observed under LD_10_ (Fig. 2E, F).

**Fig. 2:**
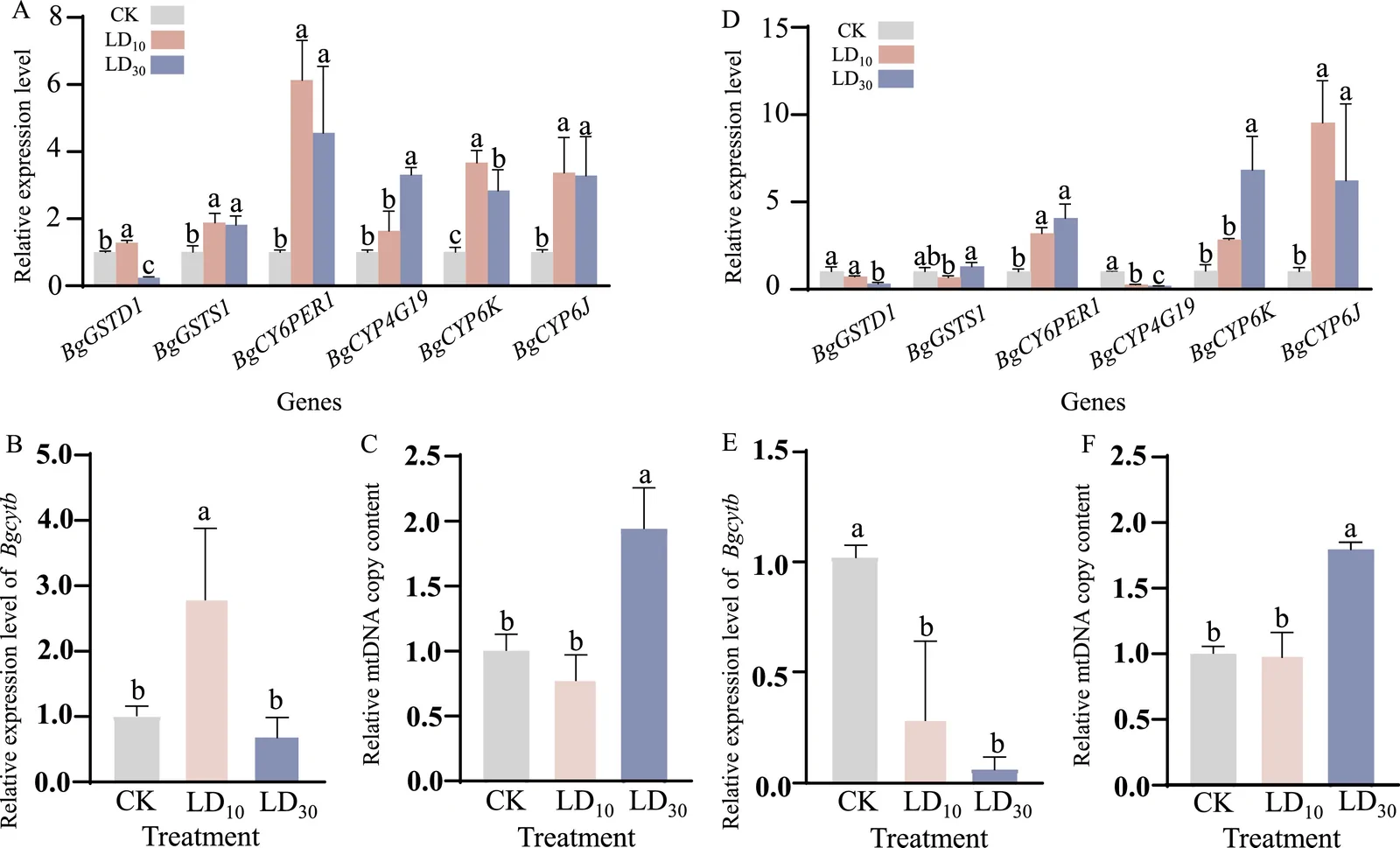
Relative expression levels of detoxification-related genes and mitochondrial indicators in the F_0_ and F_1_ generations of *Blattella germanica* following sublethal chlorpyrifos exposure. A and D show the relative expression levels of detoxification-related genes in the F_0_ and F_1_ generations, respectively. B and C show the relative expression level of *BgCytb* and mitochondrial DNA copy number in the F_0_ generation, respectively, whereas Panels E and F show the corresponding mitochondrial indicators in the F_1_ generation. Data are presented as mean ± SE. Different lowercase letters indicate significant differences among treatments within the same gene (one-way ANOVA followed by Tukey`s test, *P* < 0.05).

## 4 Discussion

Reproduction and survival are key determinants of insect fitness, and sublethal insecticide exposure can disrupt multiple life-history traits (Desneux et al., 2007; Guedes & Cutler, 2014). In the present study, sublethal chlorpyrifos exposure reduced F_0_ fecundity and altered adult survival in a sex- and dose-specific manner, while parental exposure also affected F_1_ development and reproduction. These fitness costs may reflect altered energy allocation under chemical stress (Kliot & Ghanim, 2012). Age-stage, two-sex life table analysis further revealed that parental LD_10_ exposure prolonged pre-adult development and delayed adult emergence in F_1_ offspring. Together, these findings indicate that sublethal chlorpyrifos exposure can affect fitness in F_0_ adults and is associated with changes in fitness-related traits in their F_1_ offspring.

Changes in individual growth and reproduction can influence population dynamics. In this study, LD_10_ exposure caused a sustained reduction in projected population size, whereas LD_30_ exposure mainly altered the temporal pattern of population expansion without appreciably reducing the final projected size. Although a slight compensatory reproductive response was observed under low-dose exposure, it did not translate into enhanced population growth. This contrasts with hormesis reported in some studies, where low doses stimulate reproduction or extend lifespan (Abd-Elghafar et al., 1992). Instead, our results are consistent with the fitness cost model, whereby sublethal stress suppresses population growth through cumulative reductions in survival and reproduction (Hamilton & Schal, 1990). Such discrepancies may arise from differences in exposure timing, dose range, and species-specific detoxification capacity. Notably, the effects of LD_30_ were not consistently stronger than those of LD_10_ across measured traits, a potentially non-monotonic dose-response relationship. One possible explanation is that higher exposure may induce compensatory detoxification or stress responses in surviving individuals, thereby partially buffering phenotypic effects at higher doses. (Feyereisen, 1999; Li et al., 2007). Alternatively, selective mortality under LD_30_ exposure may have increased the proportion of less susceptible individuals among the survivors, potentially contributing to the less pronounced effects observed for certain fitness-related traits compared with LD_10_. However, this explanation remains speculative, as differences in susceptibility or overall fitness among survivors were not directly assessed. Similar non-monotonic responses have been reported in insects exposed to sublethal insecticides (Guedes & Cutler, 2014; Sial et al., 2018). Thus, the stronger suppression of projected population growth under LD_10_ than LD_30_ suggests that the sublethal effects of insecticide exposure do not necessarily increase with dose.

At the molecular level, insecticide stress influences phenotypic traits through the regulation of detoxification systems and energy metabolism (Feyereisen, 1999; Li et al., 2007). In this study, we observed induced dose and generation-dependent transcriptional changes in detoxification genes. In F_1_ individuals, LD_30_ exposure increased mtDNA copy number but reduced *BgCytb* transcription, indicating a decoupling between mtDNA abundance and mitochondrial gene transcription. Cytochrome P450 and GST genes exhibited dose-dependent and intergenerational expression patterns, consistent with previous findings (Hernandez et al., 2018). Increased mtDNA copy number is often considered a compensatory response to mitochondrial dysfunction (Zeng et al., 2024); however, constrained transcriptional regulation such as limited transcription factor availability, promoter damage, or epigenetic modulation may result in incomplete functional recovery (Christmann & Kaina, 2019). Chlorpyrifos has also been shown to induce reactive oxygen species (ROS) accumulation and impair the mitochondrial electron transport chain, thereby reducing ATP production efficiency (Liu et al., 2015; Montanari et al., 2024; Pang et al., 2021). These effects may contribute to energy constraints and resource reallocation toward detoxification and repair (Salama et al., 2014), potentially affecting development and reproduction.

Several limitations should be acknowledged. First, mitochondrial function and reactive oxygen species (ROS) levels were not directly measured; therefore, the proposed detoxification-oxidative stress-mitochondrial dysfunction pathway remains hypothetical. Second, because both female and male parents were exposed to chlorpyrifos before mating, the relative contributions of maternal and paternal effects to the observed F_1_ responses could not be distinguished. Future studies should employ a factorial design that independently manipulates maternal and paternal exposure to evaluate their respective effects and potential interactions (Bonduriansky & Day, 2009). In addition, integrating metabolic assays and gene-silencing approaches would help elucidate the proposed detoxification-oxidative stress-mitochondrial dysfunction pathway and its potential contribution to fitness costs. Overall, this study provides evidence linking individual fitness, population dynamics, and molecular responses to sublethal chlorpyrifos exposure.

## Data availability statement

All data generated or analyzed during this study are included in this published article and its Supplementary Materials.

## Declaration of competing interest

All authors declare no conflicts of interest.
