# Supplementary material for: Sublethal chlorpyrifos exposure induces intergenerational fitness costs and mitochondrial alterations in Blattella germanica
Source: Curr Res Insect Sci. 2026 Oct 23;10:100136. doi: 10.1016/j.cris.2026.100136 (PMC13734963; doi:10.1016/j.cris.2026.100136)
Supplement: Supplementary file 1 [file mmc1.docx]

**Supplementary Material**

**Sublethal chlorpyrifos exposure induces intergenerational fitness costs and mitochondrial alterations in *Blattella germanica***

Xin Li ^1^, Jiang-Ling Chen ^1^, Zi-Han Xu ^1^, Jun Tao ^1^, Yi Chen ^1^, Shu-Lin He ^1^, Fei Li ^1^, Cao Zhou ^1,*^

^1^ Chongqing Key Laboratory of Vector Control and Utilization, Institute of Entomology and Molecular Biology, Chongqing Normal University, Chongqing, China

**SM1.** **Supplementary Methods**

**SM1.1 Detailed chlorpyrifos toxicity bioassay**

Adult males were selected for the toxicity bioassay because male adults exhibit relatively uniform physiological characteristics and avoid potential confounding effects associated with female reproductive status, such as ovarian development and egg production. Mortality was recorded at 72 h after treatment. Five concentrations of chlorpyrifos (0.05, 0.1, 0.15, 0.2, and 0.4 μg/μL) were used for dose-response analysis. Preliminary tests included lower and higher concentrations; however, these concentrations produced mortality responses close to 0% or 100% and were excluded because they provided limited information for LD estimation. However, the use of male adults may not fully represent sex-specific differences in chlorpyrifos susceptibility. An acetone-treated control group was included using the same anesthesia and topical application procedures as the chlorpyrifos-treated groups. Control insects received 1.0 μL acetone without chlorpyrifos. Five biological replicates of 10 males each were used for the control group (n = 50), consistent with the treated groups.

**SM1.2. Age-stage, two-sex life-table theory and parameter definitions**

The adult preoviposition period (APOP) was defined as the period from adult emergence to the first oviposition, whereas the total preoviposition period (TPOP) was defined as the period from egg hatching to the first oviposition. The effective reproductive period was defined as the period during which females continuously produced offspring.

The demographic characteristics of *Blattella germanica* were analyzed based on the age-stage, two-sex life table theory.

The age-stage-specific survival rate (*s_xj_*), where *x* is the age in days and *j* is the stage, represents the probability that a newly laid egg will survive to age *x* and stage *j*.

$$s_{xj}=n_{xj}/n_{01}$$

The age-specific survival rate (*l_x_*) and age-specific fecundity (*m_x_*) are calculated as:

$$l_{x}=\sum_{j=1}^{m} s_{xj}$$

$$m_{x}=\frac{\sum_{j=1}^{m} s_{xj}f_{xj}}{\sum_{j=1}^{m} s_{xj}}$$

where m represents the number of stages, and *f_xj_* is the age-stage-specific fecundity.

The net reproductive rate (*R*_0_) is calculated as

$$R_{0}=\sum_{x=0}^{\infty} l_{x}m_{x}$$

The intrinsic rate of increase (*r*) is estimated using the iterative bisection method based on the Euler–Lotka equation with age indexed from 0

$$\sum_{x=0}^{\infty} e^{-r\left( x+1 \right)}l_{x}m_{x}=1$$

The finite rate of increase ($\lambda$) and the mean generation time (T) are calculated

$$\lambda=e^{r}$$

$$T=\frac{\ln R_{0}}{r}$$

The age-stage-specific life expectancy (*e_xj_*), which represents the expected time that an individual of age *x* and stage *j* will live, is calculated

$$e_{xj}=\sum_{i=x}^{\infty} \sum_{y=j}^{m} s_{iy}^{'}$$

where *s'_iy_* is the probability that an individual of age *x* and stage *j* will survive to age *i* and stage *y*. The age-stage-specific reproductive value (*v_xj_*), which defines the contribution of an individual of age *x* and stage *j* to the future population, is calculated

$$v_{xj}=\frac{e^{r\left( x+1 \right)}}{s_{xj}}\sum_{i=x}^{\infty} e^{-r\left( i+1 \right)}\sum_{y=j}^{m} s_{iy}^{'}f_{iy}$$

**SM1.3. RT-qPCR conditions and primer validation**

Each 10 μL reaction contained 5 μL SYBR Green qPCR Mix, 0.5 μL of each primer, and 4 μL cDNA template. The amplification conditions were as follows: 95 °C for 10 min, followed by 39 cycles of 95 °C for 10 s, 60 °C for 15 s, and 65 °C for 5 s. The amplification efficiencies of all primer pairs were evaluated using standard curves generated from ten-fold serial dilutions of cDNA templates and are provided in Table S1. Melting curve analysis was performed after amplification to confirm the specificity of each PCR product, and all primer pairs generated a single melting peak. For RNA quality assessment, total RNA quality was evaluated using a NanoDrop spectrophotometer. Only RNA samples with concentrations above 1000 ng/μL and A260/A280 ratios ranging from 1.8 to 2.1 were used for subsequent reverse transcription.

**SM1.4. Statistical model diagnostics**

F_0_ fecundity was analyzed using a negative binomial GLMM with chlorpyrifos treatment as a fixed effect and biological replicate as a random effect, followed by Tukey-adjusted pairwise comparisons, whereas adult longevity was analyzed using Cox proportional hazards regression with biological replicate as a shared frailty term. Model diagnostics showed no overdispersion, residual non-uniformity, or influential observations in the GLMM, and the Cox models satisfied the proportional hazards assumption based on global Schoenfeld residual tests (Tables S6–S7).

**Table S1.** Primers used for RT-qPCR analysis.

| Genes | Primers | Primer sequences | Efficiency (%) | GenBank accession number |
| --- | --- | --- | --- | --- |
| *BgCYP6K1* | CYP6K1-1F | ATCTCGTATCACAGCCTGCTCT | 85% | AF281328.1 |
|  | CYP6K1-1R | GAGGTCAGGTTGGAGTGACAAC |  |  |
| *BgCYP6J1* | CYP6J1-1F | GCTGTCGCTAACCTCGCAACA | 94.8% | AF281325.1 |
|  | CYP6J1-1R | CCTCAGATGAACGGCTCTCCAG |  |  |
| *BgCYP6ER1* | CYP6ER1-1F | CGCAAGTACCCACCACTGACAA | 100% | PSN38879.1 |
|  | CYP6ER1-1R | TGACAGGGATGAGAGCTGGAGT |  |  |
| *BgGSTd1* | GSTd1-1F | TTTGCCTCTGGGAAAGCCGAG | 94.8% | AM778448.1 |
|  | GSTd1-1R | TGGTCTACAAGAGCACGCTTCT |  |  |
| *BgGSTs1* | GSTs1-1F | TGGTAAGCAGTTTGGCCTCAGT | 100% | FJ855499.1 |
|  | GSTs1-1R | ATTAGCAATGGCAGCCCTGAAG |  |  |
| *BgCYP4G19* | CYP4G19-1F | AACAGGATGAGAACGACGTTGG | 99% | AY176056.1 |
|  | CYP4G19-1R | TGGATTCCCATGAGGCAAAGGA |  |  |
| *BgCytb* | Cytb-F | AGTTAACCACATCTGCCGTGAT | 99.9% | [NC_012901.1](https://www.ncbi.nlm.nih.gov/nucleotide/NC_012901.1?report=genbank&log$=nuclalign&blast_rank=1&RID=YP7Z0GCJ014) |
|  | Cytb-R | ACCATTAGCATGTAAGGTGCGA |  |  |
| *BgEF1α* | EF1*α*-F | GCAAGGCTGAAGGCAAGTGTCT | 100% | KX228232.1 |
|  | EF1*α*-R | ACGAAGAGGCTTCTCAGTGGGT |  |  |

**Table S2**. Toxicity of chlorpyrifos against adult *Blattella germanica*

| Insecticide | Toxicity regression  equation | LD_10_ (μg/μL)  95% CI | LD_30_ (μg/μL)  95% CI | LD_50_ (μg/μL)  95%CI | df | *X^2^* |
| --- | --- | --- | --- | --- | --- | --- |
| chlorpyrifos | *y=*4.00*x+*3.10 | 0.08 (0.06 - 0.10) | 0.12 (0.10 - 0.14) | 0.17 (0.15-0.19) | 3 | 4.74 |

LD₁₀, LD₃₀, and LD₅₀ represent the chlorpyrifos concentrations causing 10%, 30%, and 50% mortality, respectively. The toxicity regression equation was established using Probit analysis. χ² indicates the chi-square value of the fitted regression model.

**Table S3** Overall effect of chlorpyrifos treatment on fecundity

| Trait | Statistical model | Test | Statistic | df | *P* value |
| --- | --- | --- | --- | --- | --- |
| Fecundity | Negative binomial GLMM | Treatment effect | *χ²=*10.919 | 2 | 0.0043 |

Fecundity was analyzed using a negative binomial generalized linear mixed model (GLMM), with chlorpyrifos treatment as a fixed effect and biological replicate as a random effect. *χ*² indicates the Wald chi-square statistic.

**Table S4** Pairwise comparisons of fecundity among treatments

| Comparison | Estimate | SE | t value | *P* value |
| --- | --- | --- | --- | --- |
| CK vs LD_10_ | -32.51 | 10.05 | -3.234 | 0.0042 |
| CK vs LD_30_ | -19.80 | 10.14 | -1.954 | 0.1224 |
| LD_10_ vs LD_30_ | 12.71 | 10.05 | 1.264 | 0.4084 |

Pairwise comparisons were performed using Tukey-adjusted estimated marginal means following the negative binomial GLMM. SE represents the standard error of the estimated difference.

Table S5. Statistical analysis of F_0_ adult longevity using Cox proportional hazards models with a shared frailty term.

| Sex | Treatment | HR | *z* | 95% CI | *P* value |
| --- | --- | --- | --- | --- | --- |
| Male | LD_10_ vs CK | 2.59 | 3.34 | 1.48–4.53 | <0.001 |
| Male | LD_30_ vs CK | 1.57 | 1.59 | 0.90–2.73 | 0.111 |
| Male | LD_30_ vs LD_10_ | 0.61 | -1.76 | 0.35 - 1.06 | 0.078 |
| Female | LD_10_ vs CK | 1.52 | 1.53 | 0.89–2.60 | 0.126 |
| Female | LD_30_ vs C K | 2.14 | 2.66 | 1.22–3.75 | 0.008 |
| Female | LD_30_ vs LD_10_ | 1.41 | 1.20 | 0.81 - 2.46 | 0.230 |

Adult longevity was analyzed using Cox proportional hazards models with biological replicate included as a shared frailty term. HR represents the hazard ratio, and CI represents the 95% confidence interval. CK indicates the acetone-treated control group, whereas LD₁₀ and LD₃₀ represent chlorpyrifos treatments causing 10% and 30% mortality, respectively.

Table S6. Diagnostic tests for the negative binomial GLMM of fecundity

| Test | Method | Result | *P* value |
| --- | --- | --- | --- |
| Dispersion test | Pearson χ² / df | Dispersion ratio = 1.02 | 0.880 |
| Residual uniformity | DHARMa KS test | D = 0.062 | 0.864 |
| Outlier detection | DHARMa outlier test | No outliers detected | 1.000 |

Model diagnostics were performed using Pearson residual dispersion testing, DHARMa residual uniformity testing, and DHARMa outlier detection. A dispersion ratio close to 1 indicates an appropriate dispersion level of the model.

Table S7. Diagnostic tests for Cox frailty models of longevity

| Model | Test | Statistic | *P* value |
| --- | --- | --- | --- |
| Male longevity | Global Schoenfeld residual test | *χ*² = 0.98, df = 2 | 0.612 |
| Female longevity | Global Schoenfeld residual test | *χ*² = 0.74, df = 2 | 0.691 |
| Male longevity | Frailty variance | θ = 0.021 | 0.517 |
| Female longevity | Frailty variance | θ = 0.034 | 0.420 |

The proportional hazards assumption was evaluated using global Schoenfeld residual tests. Frailty variance was assessed to determine the contribution of biological replicate-level variation to the Cox frailty models.

**Table S8**. Effects of sublethal chlorpyrifos exposure on developmental duration of female individuals in the F_1_ generation of *Blattella germanica*

| Treatment | Egg (d) | First-instar nymph (d) | Second-instar nymph (d) | Third-instar nymph (d) | Fourth-instar nymph (d) | Fifth-instar nymph (d) | Sixth-instar nymph (d) | Adult longevity(d) | Pre-adult period(d) | Total longevity (d) |
| --- | --- | --- | --- | --- | --- | --- | --- | --- | --- | --- |
| CK | 26.62±0.15a | 8.59±0.25a | 8.51±0.30a | 9.48±0.40a | 11.22±0.37a | 11.85±0.48a | 15.04±1.10ab | 160.51±7.53a | 91.33±1.55a | 251.85±7.16a |
| LD_10_ | 27.14±0.13b | 9.32±0.20b | 8.86±0.23a | 9.68±0.32a | 12.36±0.76a | 13.89±0.76b | 15.68±0.96a | 167.07±6.07a | 96.93±1.97b | 264.01±5.29a |
| LD_30_ | 26.82±0.21ab | 9.21±0.45ab | 9.42±0.41a | 10.35±0.57a | 12.39±0.68a | 12.07±0.64ab | 13.25±0.75b | 159.07±4.28a | 93.53±1.01ab | 252.61±4.36a |

Values are presented as mean ± SE. Different letters within the same column indicate significant differences among treatments based on paired bootstrap tests with 100,000 bootstrap resamplings (*P* < 0.05). CK indicates the acetone-treated control group, whereas LD_10_ and LD_30_ represent chlorpyrifos treatments causing 10% and 30% mortality, respectively. N1–N6 represent first- to sixth-instar nymphal stages, respectively.

**Table S9**. Effects of sublethal chlorpyrifos exposure on developmental duration of male individuals in the F_1_ generation of *Blattella germanica*

| Treatment | Egg (d) | First-instar nymph (d) | Second-instar nymph (d) | Third-instar nymph (d) | Fourth-instar nymph (d) | Fifth-instar nymph (d) | Sixth-instar nymph (d) | Adult longevity(d) | Pre-adult period(d) | Total longevity (d) |
| --- | --- | --- | --- | --- | --- | --- | --- | --- | --- | --- |
| CK | 26.82±0.15a | 8.60±0.17a | 8.52±0.30a | 10.01±0.27a | 12.21±0.38a | 11.55±0.42a | 13.79±0.68ab | 102.03±4.92a | 91.27±1.04a | 193.30±4.81a |
| LD_10_ | 27.17±0.13a | 9.60±0.28b | 9.03±0.17ab | 10.37±0.37a | 13.77±0.64b | 12.63±0.59a | 14.67±0.82a | 112.10±4.50a | 97.23±1.26b | 209.30±4.29b |
| LD_30_ | 26.83±0.20a | 9.01±0.31ab | 9.34±0.28b | 11.03±0.60a | 12.14±0.56ab | 11.31±0.75a | 12.31±0.70b | 113.44±5.37a | 91.97±1.29a | 205.41±5.16ab |

Values are presented as mean ± SE. Different letters within the same column indicate significant differences among treatments based on paired bootstrap tests with 100,000 bootstrap resamplings (*P* < 0.05). CK indicates the acetone-treated control group, whereas LD_10_ and LD_30_ represent chlorpyrifos treatments causing 10% and 30% mortality, respectively. N1–N6 represent first- to sixth-instar nymphal stages, respectively.

Figure S1. Age-stage-specific reproductive value (*v_xj_*) of the F_1_ generation of *Blattella germanica* following sublethal chlorpyrifos exposure. (A) Control; (B) LD_10_; (C) LD_30_. N1: first-instar nymph; N2: second-instar nymph; N3: third-instar nymph; N4: fourth-instar nymph; N5: fifth-instar nymph; N6: sixth-instar nymph.

Figure S2. Age-stage-specific life expectancy (*e_xj_*) of the F_1_ generation of *Blattella germanica* following sublethal chlorpyrifos exposure. (A) Control; (B) LD_10_; (C) LD_30_.

Figure S3. Projection of population growth over 200 days for the F_1_ generation of *Blattella germanica* under different sublethal chlorpyrifos treatments. (A) Control; (B) LD_10_; (C) LD_30_.
